# Rabies virus in white-nosed coatis (*Nasua narica*) in Mexico: what do we know so far?

**DOI:** 10.3389/fvets.2023.1090222

**Published:** 2023-05-09

**Authors:** Paola Puebla-Rodríguez, Cenia Almazán-Marín, Fabiola Garcés-Ayala, Emilio Rendón-Franco, Susana Chávez-López, Mauricio Gómez-Sierra, Albert Sandoval-Borja, David Martínez-Solís, Beatriz Escamilla-Ríos, Isaías Sauri-González, Adriana Alonzo-Góngora, Irma López-Martínez, Nidia Aréchiga-Ceballos

**Affiliations:** ^1^Laboratorio de Rabia, Instituto de Diagnóstico y Referencia Epidemiológicos, Departamento de Virología, Secretaría de Salud, Ciudad de México, Mexico; ^2^Facultad de Medicina Veterinaria y Zootecnia, Universidad Nacional Autónoma de México, Ciudad de México, Mexico; ^3^Departamento de Producción Agrícola y Animal, Universidad Autónoma Metropolitana, Xochimilco, Ciudad de México, Mexico; ^4^Laboratorio Central Regional de Mérida, Comité Estatal para el Fomento y Protección Pecuaria del Estado de Yucatán S.C.P., Mérida, Yucatán, Mexico

**Keywords:** rabies, white-nosed coati, spillover, host-switching, emerging variants

## Abstract

Rabies is a neglected disease that affects all mammals. To determine the appropriate sanitary measures, the schedule of preventive medicine campaigns requires the proper identification of the variants of the virus circulating in the outbreaks, the species involved, and the interspecific and intraspecific virus movements. Urban rabies has been eradicated in developed countries and is being eradicated in some developing countries. In Europe and North America, oral vaccination programs for wildlife have been successful, whereas in Latin America, Asia, and Africa, rabies remains a public health problem due to the habitation of a wide variety of wild animal species that can act as rabies virus reservoirs in their environment. After obtaining recognition from the WHO/PAHO as the first country to eliminate human rabies transmitted by dogs, Mexico faces a new challenge: the control of rabies transmitted by wildlife to humans and domestic animals. In recent years, rabies outbreaks in the white-nosed coati (*Nasua narica*) have been detected, and it is suspected that the species plays a significant role in maintaining the wild cycle of rabies in the southeast of Mexico. In this study, we discussed cases of rabies in white-nosed coatis that were diagnosed at InDRE (in English: Institute of Epidemiological Diagnosis and Reference; in Spanish: Instituto de Diagnostico y Referencia Epidemiologicos) from 1993 to 2022. This study aimed to determine whether white-nosed coatis might be an emergent rabies reservoir in the country. A total of 13 samples were registered in the database from the Rabies laboratories of Estado de Mexico (*n* = 1), Jalisco (*n* = 1), Quintana Roo (*n* = 5), Sonora (*n* = 1), and Yucatan (*n* = 5). Samples from 1993 to 2002 from Estado de Mexico, Jalisco, and Sonora were not characterized because we no longer had any samples available. Nine samples were antigenically and genetically characterized. To date, coatis have not been considered important vectors of the rabies virus. The results from our research indicate that the surveillance of the rabies virus in coatis should be relevant to prevent human cases transmitted by this species.

## Introduction

The Rabies virus (RABV) belongs to the order Mononegavirales within the family Rhabdoviridae, named after the characteristic bullet-shaped Rhabdovirus virion (1) within the *Lyssavirus* genus, and it is the cause of rabies. It is an acute infectious zoonotic disease that affects the central nervous system, causing encephalomyelitis (2). This disease is found on all continents except Antarctica, with dogs being the main transmitter (3).

Most of the RABV reservoirs belong to the order Carnivora (carnivores) from the families *Canidae, Mephitidae, Mustelidae*, and *Procyonidae* and the order Chiroptera (bats) from the families *Vespertilionidae, Molossidae*, and *Phyllostomidae*, but the circulation of RABV, *Lyssavirus* species 1, in bats is limited to New World species (4).

In countries where the urban cycle of transmission (from dog to human) has been controlled, transmission is mainly by wildlife, with bats being the main vectors (5). Currently, rabies continues to be a public health problem. It is a zoonosis that has to be solved with the “One Health” management model where a transdisciplinary approach, including social and biological sciences, the population, and governments, must work together to eliminate this disease (6).

The last case of human rabies transmitted by dogs in Mexico occurred in 2005. The decrease in the incidence of human rabies could be mainly attributed to the massive vaccination campaigns that have been carried out since the 1990s as well as the efficient epidemiological surveillance system (7). In 2019, Mexico obtained recognition from the World Health Organization (WHO) and the Pan American Health Organization (PAHO) for being the first country in the world to have met the requirements to be validated as a country free of human rabies transmitted by dogs (8).

With canine rabies currently under control, human rabies transmitted by wildlife species has become the focus of attention. From 2000 to 2019, 46 cases of human rabies transmitted by wild animals have been confirmed; of these, 90% of the attacks were from bats or skunks (8).

Mexico is ranked third among the most diverse countries in terms of mammals (9). Currently, the country has a large number of species that are capable of transmitting the RABV, both in the urban and wildlife cycles.

The Procyonidae family (order Carnivora) is a widely distributed family that includes small and medium-sized species. In Mexico, there are six different species of procyonids; one of them is the white-nosed coati (*Nasua narica*) (10) whose distribution extends from the southern United States to Colombia. In Mexico, the Procyonidae family inhabits the whole country except for Baja California and the driest part of central Mexico (11).

They inhabit all types of tropical forests, including mangroves, cloudy mountain forests, mixed coniferous forests, and oak forests (12). They are also present in highly anthropogenically disturbed areas, such as public parks in tourist cities. Coatis are social carnivores where females, young males, and newborns form groups called “bands”; however, adult males are usually solitary and only meet the band during reproduction season (13).

In spite of their wide distribution, there are few reports of rabies in coatis. In Mexico, the oldest case was diagnosed at the InDRE's Rabies Laboratory in 1993, following which no subsequent characterization was carried out. Unfortunately, there was no biological material to carry out further studies.

In 2008, a rabies outbreak with three cases of infected coatis took place at the Kabah National Park in Cancun, Quintana Roo, Mexico. The coatis were identified as being infected with the RABV antigenic variant (AgV) 9, associated with the Mexican free-tailed bat (*Tadarida brasiliensis)*. Through phylogenetic analysis, it was concluded that this AgV evolved and appeared as an independent variant despite sharing a common ancestor with AgV9 of *T. brasiliensis*, which suggests a host-switching event (14). In the United States of America (USA), the white-nosed coati is considered an infrequently reported mammalian carnivore species, and only 12 cases of rabies were registered from 1960 to 2000 (15).

The objective of this study was to characterize antigenically and genetically the cases of rabies in coatis that were registered at the Mexican Ministry of Health's Rabies Reference Laboratory to clarify the origin of the virus as well as to be able to determine the antigenic variants that circulate in white-nosed coatis.

## Materials and methods

The total number of registered cases (*n* = 13) of rabies in white-nosed coatis from a few Mexican states: Estado de Mexico (*n* = 1), Jalisco (*n* = 1), Quintana Roo (*n* = 5), Sonora (*n* = 1), and Yucatan (*n* = 5) were selected from the sample bank of the InDRE's Rabies Laboratory and the Laboratory of the Committee for Livestock Development and Protection of Merida, Yucatan (Table 1).

**Table 1:** Summary of cases of rabies in a white-nosed coatis (*Nasua narica)* in Mexico.

**#**	**Case**	**Year**	**Locality**	**State**	**Antigenic rabies virus variant**	**Sequence name**	**GenBank accesion number**
1	95161	1993	Quintana Roo	Quintana Roo	N/P	N/A	N/A
2	102655	1994	Ixtapaluca	Estado de México	N/P	N/A	N/A
3	3532	2000	Hermosillo	Sonora	N/P	N/A	N/A
4	3203	2002	N/A	Yucatan	Atypical	3203Mxcoatiyuc02	N/A
5	9021	2007	Benito Juarez	Quintana Roo	AgV9	9021Mxcoatiqroo07	N/A
6	9022	2007	Benito Juarez	Quintana Roo	AgV9	9022Mxcoatiqroo07	OM971001
7	1767	2008	Benito Juarez	Quintana Roo	AgV9	1767Mxcoatiqroo08	OM971002
8	96	2014	Tuxcueca	Jalisco	AgV8	N/A	N/A
9	1441	2019	Merida	Yucatan	Atypical	1441Mxcoatiyuc15	N/A
10	1442	2019	Sinanche	Yucatan	Atypical	1442Mxcoatiyuc15	OM971003
11	1443	2019	Tekax	Yucatan	Atypical	1443Mxcoatiyuc17	OM971004
12	25	2020	N/A	Yucatan	Atypical	25Mxcoatiyuc20	OM971005
13	368	2022	Holbox	Quintana Roo	AgV3	368Mxcoatiqroo22	N/A

N/P, Not performed; N/A, not available.

### Rabies diagnosis

The samples were diagnosed using the fluorescent antigen test (FAT) (16). The antigenic characterization was performed in 10 samples with a reduced panel of eight monoclonal antibodies (MAbs) standardized by the Center for Disease Control and Prevention (CDC) for the identification of the reservoir species (17).

### Mice inoculation test

A total of six 21-day-old mice per sample were intracranially inoculated with 0.03 ml of a 20% suspension (w/v) of homogenized brain samples in a phosphate buffer solution. The samples inoculated were 9021, 9022, and 1767 from Quintana Roo and 1441, 1442, 1443, and 25 from Yucatan. The inoculated animals were observed for 21 days and processed as described in Aréchiga-Ceballos et al. (18).

### RT-PCR and sequencing

The genetic material was extracted from nine samples of brain tissue using the Qiagen “QIAamp Viral RNA” commercial kit, according to the directions of the manufacturer. RT-PCR was used to amplify a nucleoprotein region using the following primers: 550 FW (5′ATG TGY GCT AAY TGG AGY AC 3′) and 304 RABV (5′ TTG ACG AAG ATC TTG CTC AT 3′). Amplification products and partial sequencing were performed as described in Garcés-Ayala et al. (7).

The complete RABV genome of five samples (1442, 1443, 1767, 9022, and 25) was amplified using PCR oligonucleotide primers described a previous study (19) and the SuperScript^®^ one-step RT-PCR system according to the supplier's manual (Invitrogen, Carlsbad, CA). After pooling the PCR amplicons for each sample, single-end libraries for NGS were constructed and sequenced with the Ion Torrent Personal Genome Machine (Thermo Fisher Scientific, Carlsbad, CA). Analysis of reading quality and the assemblies of the viral genome sequences were carried out using QIAGEN CLC Genomics Workbench software version 21.0.2.

### Analysis of genetic information

Sequence editing and a manual correction were performed with BioEdit software and alignment with MEGA X (20) using the MUSCLE algorithm (21). Subsequently, BLAST (Basic Local Alignment Search Tool^1^) analysis (22) from NCBI was performed.

The phylogenetic analysis was based on 148 partial nucleoprotein gene sequences of 877 nucleotides from North and Central American bats and terrestrial mammals AgVs. Multiple alignments of all sequences were performed with the Mesquite program (23) using the MUSCLE algorithm. The evolutionary model was then determined with the jModelTest 2.1.4 program (24), and the best model was GTR + G + I.

The construction of the phylogenetic tree was carried out using the BEAST 1.8.4 software (25) with the Bayesian method with 10 million generations and a burn-in of 25%. The visualization of the resulting tree was done with Figtree 1.4.3. Rambaut and Drummond (26), and editing was performed with Inkscape.

To identify the presence of possible amino acid changes between the Yucatan (*n* = 5) and Quintana Roo (*n* = 3) variants, the complete genome sequences carried out in this study (*n* = 5) were aligned with the previously described sequences from Yucatan sylvatic AgV (*n* = 4) using the MUSCLE software (27).

## Results

### Rabies virus diagnosis

The samples 95161, 102655, 3532, and 96 mentioned in Table 1 are historical records from the InDRE sample bank. Brain tissues from these samples were no longer available for this study. Nine out of thirteen samples were re-tested by FAT, and all were positive.

### Mice inoculation test

Differences in the incubation periods were observed. The clinical signs observed were as follows: hirsute hair, emaciation, and paralysis. The samples from Quintana Roo (coati AgV9) have a range between 13 and 15 days. The samples from Yucatan (Yucatan sylvatic) have a range between 19 and 20 days.

### Antigenic characterization

Four antigenic rabies virus variants were detected with the reduced panel of eight monoclonal antibodies. The samples from Quintana Roo (9021, 9022, and 1767) were AgV9, except for sample 368 from 2022, which was AgV3. The Jalisco sample [96] was AgV8. The antigenicity pattern that resulted from all samples from Yucatan (3202, 1441, 1442, 1443, and 25) did not match what was described in the panel; therefore, they were assigned AgV atypical.

### Genetic characterization

The generated phylogenetic analysis allows us to identify two different origins of the AgVs in white-nosed coatis, one related to bats and the other related to terrestrial mammals. The samples from the state of Quintana Roo (9021, 9022, 1767, and 1770) were grouped into an independent clade belonging to the Mexican free-tailed bat *T. brasiliensis*. Sequences from white-nosed coatis from 2007 to 2008 were clustered in a single sub-clade, while the viruses isolated from the bat *T. brasiliensis* were clustered in an independent sub-clade, indicating a divergent event between the virus from bats and the coati virus.

Sample 368 from a coati (2022) is also clustered into a clade of viruses with bat origin, but this one was grouped with the sequences related to the common vampire bat, *Desmodus rotundus*. To date, this is the first report of a vampire bat AgV infection in coatis in the region.

**Figure 1:**
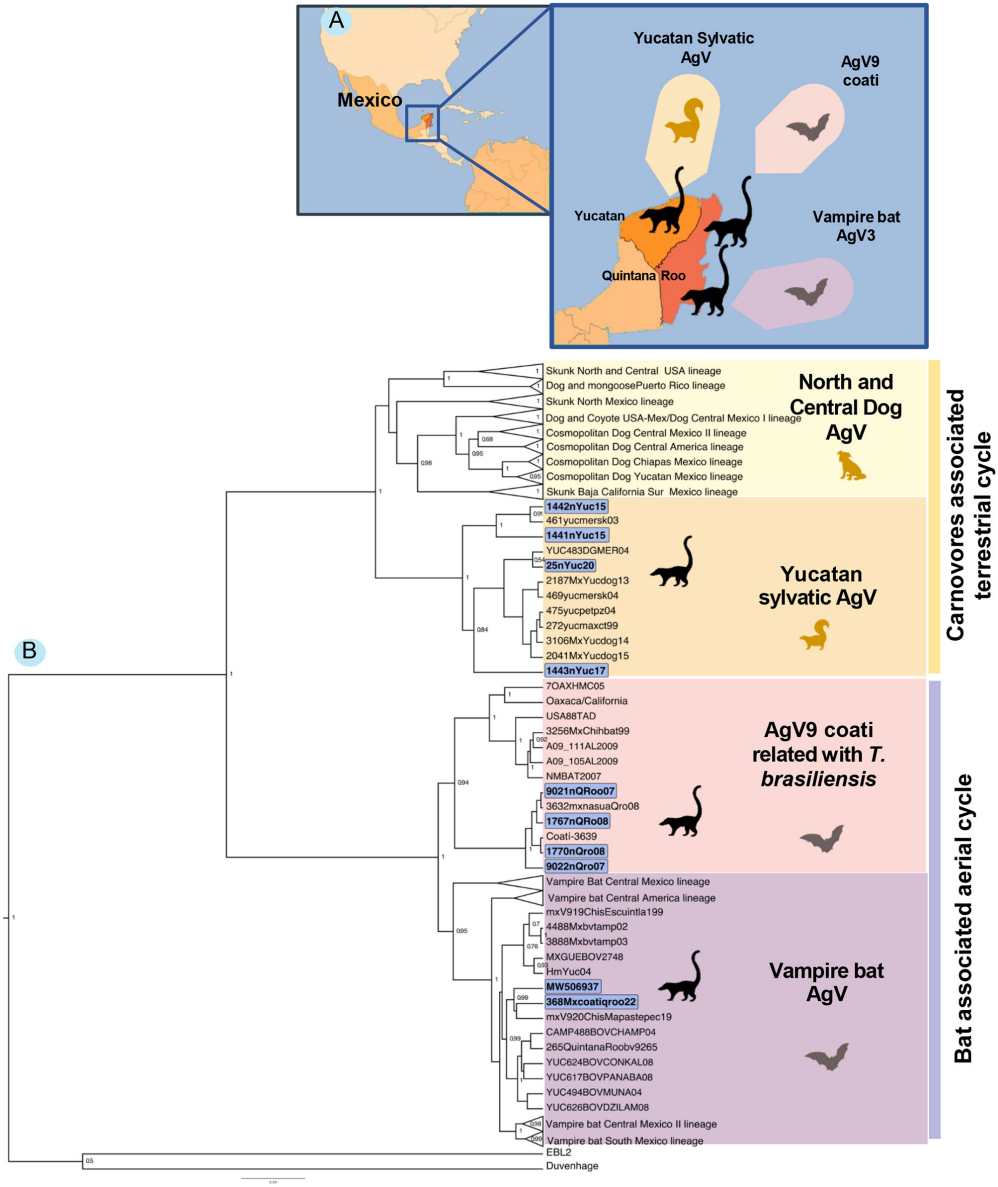
**(A)** Location of the coati samples and their association with terrestrial mammals and bats. **(B)** Bayesian phylogenetic tree of the white-nosed coati (*Nasua narica*) rabies cases in the Yucatan Peninsula (The states of Yucatan and Quintana Roo) including other AgV isolated from different reservoir species.

In the terrestrial mammals' clade, the Yucatan samples (1441, 1442, 1443, and 25) are grouped in a lineage that was previously described as “Yucatan sylvatic,” clustered with viruses isolated from dogs, skunks, and other wild-living mammals (Figure 1).

The sample sequences from the state of Yucatan are clustered in a single lineage with two sub-clades. One study grouped the viruses from 2015 with a virus isolated from a skunk in 2003. However, the other sub-clade grouped the last reported cases of RABV in dogs (in 2004, 2014, and 2015) in Yucatan. Some of these dogs and a lowland paca (*Cuniculus paca*) were attacked by skunks (unknown species). This lineage is part of the terrestrial sylvatic cycle of rabies in the state of Yucatan with a canine origin, but, at present, some other species (like skunks) are acting as active transmitters to non-vaccinated dogs and other wild-living species.

The analysis of the composition of the amino acids of the full genome sequences performed for the five proteins of the RABV showed that between the coati AgV9 and Yucatan sylvatic, 28 changes were detected in the nucleoprotein, 51 changes in the phosphoprotein, 19 changes in the matrix protein, 79 changes in the glycoprotein, and 112 in the large or polymerase protein (Table 2).

**Table 2:** Summary of the comparison of the amino acid differences of the 5 RABV proteins found between the coati AgV9 and Yucatan sylvatic AgV.

**Protein**	**Position**	**Amino acid in samples from Quintana Roo**	**Amino acid in samples from Yucatan**	**Type of change**
***N***	3	T	A	NP → NH
	36	L	S	NH → NP
	42	T	I	NP → NH
	98	R	Q	B → NP
	106	D	S	A → NP
	115	N	D	NP → A
	135	A	S	NH → NP
	367	Q	K	NP → B
	377	A	T	NH → NP
	379	T	A	NP → NH
	433	T	A	NP → NH
***M***	87	Q	H	NP → B
	148	A	T	NH → NP
	154	E	Q	A → NP
	190	R	G	B → NP
***G***	3	L	S	NH → NP
	109	I	T	NH → NP
	132	Q	H	NP → B
	174	P	S	NH → NP
	175	G	R	NP → B
	176	G	E	NP → A
	201	E	N	A → NP
	225	V	T	NH → NP
	261	T	A	NP → NH
	273	A	S	NH → NP
	322	Y	H	NP → B
	367	K	G	B → NP
	368	E	G	A → NP
	375	D	N	A → NP
	389	N	H	NP → B
	423	T	A	NP → NH
	466	G	A	NP → NH
	494	G	E	NP → A
	504	P	S	NH → NP
	507	R	G	B → NP
***P***	29	A	N	NH → NP
	55	R	Q	B → NP
	57	Q	R	NP → B
	61	D	G	A → NP
	63	P	S	NH → NP
	68	S	E	NP → A
	78	Q	R	NP → B
	86	A	G	NH → NP
	90	A	S	NH → NP
	112	G	E	NP → A
	130	A	T	NH → NP
	158	K	T	B → NP
	160	A	T	NH → NP
	161	S	P	NP → NH
	165	A	E	NH → A
	168	P	S	NH → NP
	171	V	T	NH → NP
	177	T	V	NP → NH
	189	A	T	NH → NP
	241	S	A	NP → NH
	248	A	T	NH → NP
	270	H	N	B → NP
***L***	4	P	S	NH → NP
	8	F	Y	NH → NP
	21	S	P	NP → NH
	24	N	A	NP → NH
	25	P	S	NH → NP
	46	S	P	NP → NH
	48	Q	R	NP → B
	55	K	E	B → A
	61	L	Y	NH → NP
	104	Y	H	NP → B
	107	Y	H	NP → B
	118	T	A	NP → NH
	122	Q	H	NP → B
	139	E	G	A → NP
	170	I	T	NH → NP
	203	A	T	NH → NP
	210	D	N	A → NP
	217	K	Q	B → NP
	285	H	Q	B → NP
	346	M	K	NH → B
	426	S	L	NP → NH
	430	S	A	NP → NH
	741	Q	P	NP → NH
	933	N	H	NP → B
	1092	T	P	NP → NH
	1172	M	T	NH → NP
	1255	A	S	NH → NP
	1356	R	Q	B → NP
	1475	A	S	NH → NP
	1884	H	Y	B → NP
	1889	A	T	NH → NP
	1996	S	A	NP → NH

The protein analysis allows a clear difference between cycles of coati AgV9 and Yucatan Sylvatic AgV. The genomes of this last one present high homogeneity between amino acid sequences for each RABV protein and without significant changes between the sequences isolated from different species or years.

According to the changes that Garces-Ayala et al. (27) mentioned having found in position 379, the finding of the change for alanine is described, which had also previously been found in the genomes carried out in this research. This amino acid change is shared with sequences from skunks. In the same way, the presence of lysine in position 367 had previously been reported in sequences from the state of Yucatan, where the probable reservoir was a skunk species (28), which coincides with the report mentioned by Garcés-Ayala et al. (27), in which at least one of the dogs from which the virus was isolated was attacked by a skunk.

**Figure 2:**
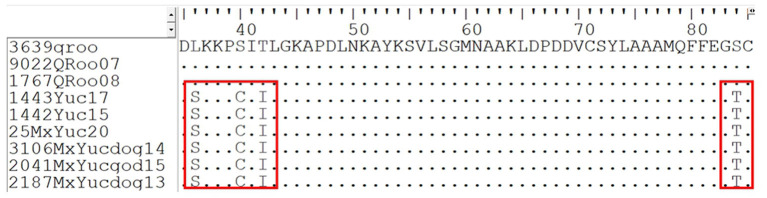
Fragment of the alignment of the nucleoprotein gene of domestic dog (*Canis lupus familiaris*) and white-nosed coatis (*Nasua narica*) sequences, marked in red, shows the punctual sites where there is a substitution of amino acids present only in the sequences belonging to the lineage of Yucatan sylvatic.

In the specific case of the nucleoprotein, it can be observed that amino acid substitution is present only in those isolated sequences from the state of Yucatan, which corresponds to the Yucatan Sylvatic lineage, as shown in Figure 2. At position 36, it could be observed that all the sequences present serine; at position 40, they present cysteine; at position 41, they present isoleucine; and at position 84, they present threonine. These substitutions tend to be exclusive to the Yucatan Sylvatic lineage since they have been present and conserved in all the sequences that have been isolated over a period of 7 years (2013–2020). These results provide the support that this lineage is endemic to the terrestrial cycle in the state of Yucatan.

## Discussion

In this study, four AgVs were detected in white-nosed coatis from Mexico. Two of them originated in bats: coati AgV9 associated with *Tadarida brasiliensis* and coati AgV3 associated with from *Desmodus rotundus*. The other two are terrestrial and are related to skunks (AgV8 and the Yucatan sylvatic variant).

The AgV8 from Jalisco was not available for genetic characterization and therefore was not included in the analyses performed in this study. However, recent studies on AgV8 have shown that it can be detected and harbored by skunks and vampire bats; therefore, further studies are needed to distinguish when rabies is transmitted by which species.

The other three variants are coati AgV9 and AgV3 from Quintana Roo and the Yucatan sylvatic variant. However, although coati AgV9 and the Yucatan sylvatic variant were found in two adjoining states, to date, there has been no evidence of geographical overlapping. It appears to be restricted to the state in which they have been detected, and even when they are infecting the same species, they are not phylogenetically related.

The geographic distribution of the virus plays an important role in virus transmission. Even when some hosts have a wide distribution, the virus variant is usually more restricted (29, 30). This implies a specific virus ecological niche (31), which could explain the geographic restriction of AgV9 and Yucatan sylvatic.

Regional differences may be influenced by the disease transmission dynamics in each state since cross-species transmission of rabies among terrestrial animals may be influenced by animal susceptibility, population density, animal behavior, niche overlap, landscape characteristics, human population distribution, and encroachment into wildlife habitats, among other factors (32).

Another piece of evidence supporting the independent origin of these two variants is the incubation period in mice. The samples from Quintana Roo (coati AgV9) have a range between 13 and 15 days, while those from the Yucatan sylvatic AgV have a range between 19 and 20 days. This difference in the incubation period also has implications for virus pathogenicity. Longer incubation periods have shown an increased transmission rate since the virus is more likely to be shed in saliva (33).

Due to two rabies outbreaks in white-nosed coatis with the same AgV in Quintana Roo in 2007 and 2008, we hypothesized that coati AgV9 evolved by host-switching from insectivorous bats. Nevertheless, the bat reservoir species origin of this virus has not yet been determined since the natural distribution of *T. brasiliensis*, which is associated with AgV9 in Mexico, does not include the Yucatan Peninsula (34). Therefore, we consider bats in the Molossidae family to be the most probable origin, but the species that harbors this AgV remains to be identified due to a lack of RABV surveillance in bat species in this region of the country.

Thus far, white-nosed coatis have not been considered important RABV reservoirs anywhere; however, our results suggest that a new lineage was established in the coatis of Quintana Roo; therefore, surveillance of this species should be relevant in the control of rabies in Mexico. Interestingly, the last case of rabies in a white-nosed coati in Quintana Roo was transmitted by *D. rotundus*, and the coati AgV9 has not been detected in recent years. Since oral vaccination campaigns for wild species are not carried out in Mexico, we can rule out that this variant is extinct due to vaccination.

There could be two reasons for which Coati AgV9 has not been detected in recent years: (1) The lack of rabies surveillance in the region led to non-detected positive cases, and (2) the disease became extinct. This phenomenon has been described for specific localities in cattle and dogs without any specific prevention measures implemented (35, 36).

Some characteristics of the white-nosed coatis support the hypothesis of a self-limited RABV outbreak caused by AgV9 since coatis may lack intrinsic competency to contribute to transmission and may be an emergent reservoir species of RABV, failing to establish and maintain intra-specific transmission. Several examples prove that RABV persists in numerous species-specific cycles that rarely sustain transmission in alternative species (37–40).

A study that used host traits to predict reservoir host species of RABV detected that having short lifespans and reproducing rapidly were among the most important traits for being a carnivore RABV reservoir. However, other species besides coatis of the Procyonidae family, the raccoon (*Procyon lotor*) and the kinkajou (*Potos flavus*), with the traits mentioned, were predicted to be non-reservoirs (41). This contrasts with the evidence collected in the last 10 years, which proves that new AgVs of the bat origin have been described in kinkajous in Peru and Brazil (42, 43), and raccoons are a well-known RABV reservoir in the USA and Canada.

Clustering may be a reason for the reduction of pathogen transmission through the buildup of immune individuals (44). The white-nosed coati presents a unique social structure within the order Carnivora (45); adult males are solitary, while females and males under 2 years of age live in groups called bands of between 5 and 26 individual animals (13).

In the rabies outbreak of 2008, one out of six coatis (and the only male) sampled showed 1.3 IU/ml of rabies virus neutralizing antibodies, while five females were all naïve. This may suggest that males may be more prone to non-lethal infection given their more solitary social behavior and acquired antibodies against RABV (14). Sex bias in rabies susceptibility has been reported in bat-eared foxes (*Otocyon megalotis*), with higher energy stress in females being attributed to their increased susceptibility to RABV (46).

The presence of antibodies against RABV in non-vaccinated coatis was previously described in 1998 in a study performed at Chamela, Jalisco, Mexico (47) and in São Paulo, Brazil. In this study, two out of two brown-nosed coatis (*Nasua nasua*) tested positive for RVNA (48).

Transmission of diseases such as rabies that require direct contact is heavily influenced by the density of susceptible animals and their contact rate with infected animals (49). There is a lack of studies regarding the density of white-nosed coatis populations in the Yucatan Peninsula; however, recent data from Quintana Roo's Ministry of Ecology and Environment (2020) indicates that, at Kabah Park, the coati density is 0.361/km (50). In 2007 and 2008, when the rabies outbreaks took place, the population density was ignored; therefore, the transmission rate of the coati variant could not be established.

Social behavior has the potential to alter disease transmission dynamics (32). In coatis, this behavior includes allogrooming, allonursing, and babysitting. This may explain why the three cases of the outbreak in 2008 were females, since social networks with high connectivity and individual animals' frequent association with each other may facilitate the spread of rabies, as has been described in raccoon populations (51).

The rabies outbreaks in 2007 and 2008 at Quintana Roo have proved that, even when the coati AgV9 has not been recently detected, there is a risk of the emergence of new reservoir species. Since ecological niches are shared by bats and terrestrial mammals in some tropical and subtropical areas of Mexico, both cycles are present (52) with the potential for the emergence of a new RABV reservoir.

By contrast, the Yucatan sylvatic variant was previously detected in non-vaccinated dogs attacked by skunks (27). Since these viruses share a canine origin, it makes them prone to return to dogs, where the disease can easily become enzootic again (53). Therefore, vaccination campaigns must continue to avoid the reintroduction of either skunks or coatis to non-vaccinated dogs.

The fact that this atypical AgV from Yucatan has been detected in domestic dogs, skunks (unknown species), white-nosed coatis, and other wild mammals such as deer (*Odocoileus virginianus*) and lowland pacas (28) may indicate that the terrestrial RABV cycle in this state can be more complex.

To date, skunks tend to act as active transmitters, even when they are considered aposematic species; however, the rate of transmission has not been determined, and there are many unknown facts; for instance, the skunk species that act as spreaders have not yet been identified.

Host contact rates can be influenced by variables such as habitat quality, host behavior, and local carrying capacity (54). In Yucatan, evidence suggests that habitat quality and spatial overlap can have a greater impact on cross-species transmission by generating competition for similar resources, which increases the potential for pathogen spillover (55).

In fact, on the Yucatan Peninsula, Quintana Roo is the state where the natural geocomplexes are best preserved, while the state of Yucatan has more altered geocomplexes. The modification of the more intense landscapes was caused by a greater anthropogenic impact due to urbanization, mining and industrial activities, cattle, poultry, pig farming, tourism, and electricity-generating infrastructure (56).

Surveillance should be improved to define if a single skunk species is acting as a vector/reservoir or if other wild-living species are involved. Future studies will be required to identify which of the skunk species present in the state of Yucatan is the reservoir of this AgV.

The amino acid analysis performed in this study supports the idea that the Yucatan sylvatic AgV has a canine origin; however, it has now been introduced into other wild-living mammals. Evidence indicates that this variant is maintained in populations of skunks, at present, with occasional spillovers to other wild animals, such as coatis and lowland pacas, and domestic animals, such as dogs and cats.

To date, white-nosed coatis have not been involved in rabies human cases in Mexico, but they inhabit ecological parks, which could place them in direct contact with humans and domestic animals, making them a potential source of RABV infection. According to data from the Rabies and other Zoonoses Program in the state of Yucatan, every year, an average of 10 post-exposure prophylaxis treatments are applied after human contact or aggression by white-nosed coatis. The population must be informed about this potential risk when coming into direct contact with this species, particularly in tourist areas where it is common for white-nosed coatis to approach humans and ask for food.

## Data availability statement

The datasets presented in this study can be found in online repositories. The name of the repository and accession numbers can be found below: NCBI; OM971001-OM971005.

## Ethics statement

Ethical review and approval was not required for the animal study because it is part of the rabies surveillance system performed at the Ministry of Health in Mexico, for which ethics approval is not required. The methods are described in the Mexican Legislation NOM-011-SSA2-2011 for the control and prevention of human rabies, in dogs and cats.

## Author contributions

Conceptualization: NA-C and PP-R. Methodology: PP-R, FG-A, CA-M, SC-L, MG-S, DM-S, AS-B, BE-R, IS-G, and AA-G. Formal analysis: PP-R, FG-A, CA-M, and NA-C. Investigation: NA-C, FG-A, AS-B, and ER-F. Resources and funding acquisition: NA-C and IL-M. Data curation: FG-A, NA-C, and CA-M. Writing—original draft preparation: NA-C. Writing—review and editing: PP-R, NA-C, and ER-F. All authors have read and agreed to the published version of the manuscript.

## Footnotes

^1^ https://blast.ncbi.nlm.nih.gov/Blast.cgi
